# Identification and Characterization of Two Bibenzyl Glycosyltransferases from the Liverwort *Marchantia polymorpha*

**DOI:** 10.3390/antiox11040735

**Published:** 2022-04-08

**Authors:** Rui-Lin Xiong, Jiao-Zhen Zhang, Xin-Yan Liu, Jian-Qun Deng, Ting-Ting Zhu, Rong Ni, Hui Tan, Ju-Zheng Sheng, Hong-Xiang Lou, Ai-Xia Cheng

**Affiliations:** 1 Key Laboratory of Chemical Biology of Natural Products (Ministry of Education), School of Pharmaceutical Sciences, Cheeloo College of Medicine, Shandong University, Jinan 250012, China; xiongruilin@mail.sdu.edu.cn (R.-L.X.); zhangjiaozhen@sdu.eud.cn (J.-Z.Z.); dengjianqun@mail.sdu.edu.cn (J.-Q.D.); tingtingzhu@mail.sdu.edu.cn (T.-T.Z.); nirong@mail.sdu.edu.cn (R.N.); tanhui@mail.sdu.edu.cn (H.T.); shengjuzheng@sdu.edu.cn (J.-Z.S.); 2 Department of Pharmacy, Qilu Hospital of Shandong University, Jinan 250012, China; liuxinyan@qiluhospital.com

**Keywords:** *Marchantia polymorpha*, *O*-glucosyltransferase, bibenzyls, biosynthesis, enzymatic catalysis

## Abstract

Liverworts are rich in bibenzyls and related *O*-glycosides, which show antioxidant activity. However, glycosyltransferases that catalyze the glycosylation of bibenzyls have not yet been characterized. Here, we identified two bibenzyl UDP-glucosyltransferases named *Mp*UGT737B1 and *Mp*UGT741A1 from the model liverwort *Marchantia polymorpha*. The in vitro enzymatic assay revealed that *Mp*UGT741A1 specifically accepted the bibenzyl lunularin as substrate. *Mp*UGT737B1 could accept bibenzyls, dihydrochalcone and phenylpropanoids as substrates, and could convert phloretin to phloretin-4-*O*-glucoside and phloridzin, which showed inhibitory activity against tyrosinase and antioxidant activity. The results of sugar donor selectivity showed that *Mp*UGT737B1 and *Mp*UGT741A1 could only accept UDP-glucose as a substrate. The expression levels of these *Mp*UGTs were considerably increased after UV irradiation, which generally caused oxidative damage. This result indicates that *Mp*UGT737B1 and *Mp*UGT741A1 may play a role in plant stress adaption. Subcellular localization indicates that *Mp*UGT737B1 and *Mp*UGT741A1 were expressed in the cytoplasm and nucleus. These enzymes should provide candidate genes for the synthesis of bioactive bibenzyl *O*-glucosides and the improvement of plant antioxidant capacity.

## 1. Introduction

Liverworts belong to a sub-group of the non-vascular bryophytes and produce a variety of natural metabolites including bibenzyls and flavonoids, which show excellent antioxidant activity [1,2]. Flavonoids are widespread in nature, while bibenzyls are distributed exclusively in liverworts [3] and some vascular plants such as Orchidaceae [4]. Flavonoids contain a C6-C3-C6 backbone and have been classified into several subgroups, namely flavanones, dihydroflavonols, flavones, flavonols, flavan-3,4-diols, flavan-3-ols and anthocyanins according to their oxidation status and substitution patterns of the core skeleton [5]. Bibenzyls contain a C6-C2-C6 skeleton. Both bibenzyls and flavonoids are derived from the phenylpropanoids pathway and share several similar upstream steps [6]. Flavonoids and bibenzyls not only play significant physiological roles in plants, but also have important medicinal properties, such as antioxidant, antibacterial and anti-inflammatory activity [7,8,9]. Various modifications of these compounds, including glycosylation, acylation and methylation, make the structure of natural products more diversified. Glycosylation can significantly affect the solubility, stability and toxicity of the compounds [10] and is usually essential for the storage, transportation and maintaining metabolic homeostasis of polyphenols [11]. 

In plants, the glycosylation of natural products is usually catalyzed by the uridine diphosphate-dependent glycosyltransferases (UGTs). UGTs belong to the large glycosyltransferases 1 family (CAZy database) [12], which contain a Plant Secondary Product Glycosyltransferase (PSPG) motif at the C-terminus. The PSPG domain comprises 44 conserved amino acids and serves as a UDP-sugar binding site [13,14]. UDP-sugars, as the supplier of glycosyl residues of various plant glycosides, mainly include UDP-glucose, UDP-galactose, UDP-glucuronic acid, UDP-xylose and UDP-rhamnose. 

Glycosylation of natural products in plants not only increases molecular diversity, but also plays an important physiological role in plant growth. Studies have shown that the overexpression of UGT79B2/B3 and UGT87A2 in Arabidopsis significantly enhances the tolerance of plants to low temperature, drought and salt stress. Therefore, it was demonstrated that the accumulation of glycosides confers significant abiotic stress tolerance in plants [15,16]. In addition, many important small-molecule compounds in plants are usually toxic and unstable, so they rarely accumulate in plant as aglycones. Thus, glycosylation in plants is also a way to reduce the toxicity of products. For example, phenylpropanes are important biosynthetic precursors of lignin, and their aglycones are toxic to plants, so they usually exist in the form of glycosides, which are often associated with plant resistance to fungal infections and bacterial invasion [17,18]. 

During recent years, many flavonoid glycosyltransferases from various plants, including *Glycyrrhiza uralensis* [19], *Vitis vinifera* [20], apple [21] and *Camellia sinensis* [22], have been functionally characterized. Moreover, most of the identified flavonoid glycosyltransferases showed high catalytic activity for flavones and flavonols to form their corresponding 7-*O*-glycosides or 3-*O*-glycosides. Phloretin belongs to dihydrochalcone, which usually exists in plants in the form of glycosides including phloridzin and trilobatin. Phloridzin showed antioxidant and anti-aging activities in *Drosophila melanogaster* [23]. Several plant phloretin glycosyltransferases, which catalyze the formation of phloridzin and trilobatin, have been characterized [24,25]. However, specific and efficient phloretin-4-*O-*glycosyltransferase is still lacking. Phloretin-4-*O-*glucoside as a new dihydrochalcone glycoside, was firstly isolated from the stems of *Homalium stenophyllum* in 2017 and showed inhibitory activity against tyrosinase and thus has antioxidant activity [26,27]. Bibenzyl glucosides usually have the effect of anti-melanin production. For example, dihydroresveratrol-4-*O*-glucoside showed inhibiting activity in B16F0 melanoma cells [28]. However, the UGTs using bibenzyls as substrates have not yet been functionally characterized.

Liverworts are the first plant lineage to produce flavonoids and are rich in bibenzyls. Therefore, it is of great significance to study the role of UDP-glycosyltransferase in the biosynthesis of flavonoid and bibenzyl glycosides in liverworts. To date, only five flavonoid glucosyltransferases have been characterized in liverworts, which catalyze different flavonoids to form their corresponding 7-*O*-glycosides or 3-*O*-glycosides [29,30]. Moreover, the flavonoid or bibenzyl glucosyltransferases in the model liverwort *Marchantia polymorpha* have not been characterized. In order to broaden our understanding of the enzymes responsible for bibenzyls or flavonoids glycosylation in liverworts, in the present investigation, we screened the *M. polymorpha* genomes and characterized two UGTs with different catalytic characteristics and substrate selectivity *in vitro*. *Mp*UGT741A1 was highly specific for the bibenzyl lunularin. *Mp*UGT737B1 accepted bibenzyls, phloretin and phenylpropanoids as substrates and catalyzed phloretin to form the rare natural product phloretin-4-*O*-glucoside. The bibenzyl glycosyltransferases, which can be used as candidate genes for the synthesis of antioxidant compounds and improving plant antioxidant capacity, were identified from liverworts for the first time.

## 2. Materials and Methods

### 2.1. Plant Materials and Chemicals

*Marchantia polyraorpha* was collected in Leshan, Sichuan, China and tissue cultured in the laboratory of Shandong University. The *M. polymorpha* and *Nicotiana tabacum* L. were grown in the plant growth chamber at a temperature of 24 °C and a 16/8 h photoperiod. The seven-week-old thallus of *M. polymorpha* were collected, immediately frozen in liquid nitrogen, and stored at −80 °C for subsequent experiments.

Unless otherwise stated, chemical standards were purchased from Chengdu Must Bio-technology (Chengdu, China). Caffeoyl aldehyde, 5-OH coniferyl alcohol and 5-OH coniferaldehyde were all synthesized in the laboratory using existing methods [31]. UDP-glucose, UDP-galactose and UDP-glucuronic acid were purchased from Sigma-Aldrich (St. Louis, MO, USA).

### 2.2. Sequence Alignment and Phylogenetic Analysis

Using UDP-glycosyl transferase as the query words, combined with sequence alignment and blast, two putative UGTs were selected from the *M. polymorpha* genome data (GenBank accession number: PRJNA53523). Their deduced polypeptide sequence was aligned with the identified UDP-glucosyltransferase *Vv*GT1 (*Vitis vinifera* GT, AAB81682) and *Pa*GT2 (*Phytolacca americana* GT, AB368371) using DNAMAN v7.0.2 software (Lynnon Biosoft, Quebec, QC, Canada) and a phylogenetic analysis was performed using MEGA v5.0.1 software (http://www.megasoftware.net, accessed on 26 February 2020), based on the neighbor-joining method [32].

### 2.3. RNA Extraction and cDNA Cloning

Total RNA was extracted and purified from the thallus of *M. polymorpha* using the cetyltrimethylammonium bromide (CTAB) method [33]. The extracted RNA was converted to cDNA using a PrimerScriptRT Master Mix kit (including gDNA eraser) (Takara, Kyoto, Japan) following the manufacturer’s instructions. The open reading frames (ORFs) of the *Mp*UGTs were amplified from the cDNA template using *ApexHF* HS DNA Polymerase FS Master Mix (Accurate Biotechnology, Changsha, China). The amplified fragments were digested with the corresponding restriction enzyme, and then inserted into pET32a.

### 2.4. Heterologous Expression and Purification of Recombinant UGT Proteins

The ORFs of *M. polymorpha* UGT genes were cloned into expression vector pET32a (+) (Novagen, Malaysia) with a Trx-S-His tag at the N-terminus. The relevant primer sequences are listed in Table S1. *E. coli* strain BL21 (DE3) competent cells (Novagen) were transformed with recombinant plasmids pET32a*Mp*UGT737B1 and pET32a*Mp*UGT741A1 and the empty pET32a plasmid. The expression and purification of the recombinant protein were performed according to the previously reported procedure [34]. 

### 2.5. Enzyme Assay and Product Identification

To demonstrate the *Mp*UGTs’ activity and identify their substrate selectivity, these enzymes were reacted with various flavonoids, bibenzyls and phenylpropanoids. The total 150 μL reaction system included 200 mM Tris-HCl buffer (pH 7.5), 1 mM dithiothreitol (DTT), 15 μg recombinant protein, 1 mM UDP-sugar donor (UDP-glucose or UDP-galactose or UDP-glucuronic acid) and 100 μM of a series of sugar acceptors incubated at 30 °C for 1 h. The reaction was generally extracted twice with 150 μL of ethyl acetate. After centrifugation for 5 min, the organic phase was evaporated and the residue was dissolved in 100 μL methanol for high performance liquid chromatography (HPLC) analysis and LC-MS analysis. When UDP-glucuronic acid or UDP-galactose was the sugar donor, the reactions were terminated by adding an equal volume of methanol, followed by centrifugation at 12,000 rpm for 20 min. Then the supernatant was analyzed by HPLC. The negative control incubations replaced the recombinant protein with the protein expressed by the empty pET32a plasmid.

To identify the enzymatic products of *Mp*UGT737B1 using phloretin as a substrate, a large-scale enzymatic assay was performed in which the *Mp*UGT737B1 reaction was scaled up to 150 mL and kept at 30 °C for 6 h. The products were extracted in 200 mL EtOAc, and then the organic phase was evaporated. The residue was dissolved in methanol, and separated by reversed-phase HPLC. Product structures were identified by combining mass spectrometry (MS), nuclear magnetic analysis ^1^H NMR, heteronuclear singular quantum correlation (HSQC) and ^1^H-^1^H correlation spectroscopy (^1^H-^1^H COSY).

To explore the effect of reaction temperature on enzyme activity, the reaction was conducted across a 20–55 °C range with a pH value of 7.5. To test the pH sensitivity of the reaction, 400 mM MES (pH 5.0–6.0), Tris-HCl (pH 6.5–8.0) or potassium phosphate (pH 8.5–9.5) were used as buffers to perform the reaction at 30 °C. To test the effect of divalent metal ions on enzyme activity, metal ions Mg^2+^, Ca^2+^, Mn^2+^, Ni^2+^, Fe^2+^ and Cu^2+^ with a final concentration of 5 mM were added to the reaction, respectively, and the activity test was performed without metal ions or adding EDTA as a control.

For kinetic analysis of the recombinant *Mp*UGT737B1 and *Mp*UGT741A1 proteins, the concentrations of the phloretin and lunularin substrates were constructed from 5 to 300 μM in reaction mixtures. The assays were performed in triplicate for 12 min at the optimal pH and temperature. Then the reaction was terminated and the product was analyzed as described above. Subsequently, the *V_max_* and *K_m_* values were calculated using the GraphPad Prism 8.0.2 software (La Jolla, CA, USA).

### 2.6. In Vivo Functional Analysis of MpUGT737B1 in Escherichia coli

The recombinant *E. coli* MpUGT737B1 strain was inoculated into 4 mL of LB medium containing 100 μg/mL ampicillin and grown at 37 °C for 16–18 h. The culture was inoculated into 50 mL of the same medium and incubated under the same conditions until the OD600 reached about 0.5. Then 0.5 mM IPTG was added to induce protein expression. After culturing at 16 °C for about 5–6 h, each aliquot of 10 mL was fed with the corresponding substrates (100 μM of phloretin or dihydroresveratrol). The metabolites were collected by extracting twice with equal volumes of ethyl acetate from aliquots of 0.5 mL cultures sampled regularly. The organic phase was evaporated under the pump and suspended in 100 μL methanol for HPLC analysis.

In order to optimize the feeding conditions, the LB was replaced with two different media, M9 minimal and Terrific Broth (TB), to screen for the most suitable medium with phloretin as the substrate. Subsequently, in the optimal medium, different final concentrations of substrates in the range of 75–300 μM were used for feeding analysis. All samples were taken 18 h after the addition of phloretin. 

### 2.7. HPLC Analysis and LC-MS Analysis of the Product

In this study, the samples were analyzed by HPLC-MS at a flow rate of 0.5 mL/min through Agilent Zorbax SB-C18 column (150 mm × 4.6 mm, 5 μm). Methanol (A) and water containing 0.1% formic acid (B) were used as mobile phases. Method (a) was used for flavonoids, Method (b) for bibenzyls and Method (c) for phenylpropanoids. Method (a): 0–20 min, 35–65% A; 20.1–25 min, 100% A; 25.1–30 min, 35% A. Method (b): 0–10 min, 30–45% A; 10–20 min, 45–80% A; 20.1–25 min, 100% A; 25.1–30 min, 30% A. Method (c): 0–20 min, 15–60% A; 20–21 min, 60–100% A; 21–25 min, 100% A; 25–27 min, 100–15% A; 27–30 min, 15% A. Samples were prepared by HPLC at a flow rate of 1.5 mL/min through anEclipse XDB-C18 column (250 mm × 9.4 mm, 5 μm). The HPLC separation condition was that 57% A and 43% B were eluted equivalently, and examined at 280 nm. The conversion percentages were calculated based on the peak areas of glycosylated products and substrates analyzed by HPLC.

### 2.8. Expression Patterns of the MpUGTs’ Response to UV Treatment

Seven-week-old thallus of *M. polymorpha* were exposed to ultraviolet light for 10 min. Samples were taken after 6, 12, 24, 36, 48 and 60 h and immediately frozen in liquid nitrogen and stored at −80 °C. The control group was the sample without UV treatment. Then, based on these materials, expression analysis was performed by qRT-PCR with SYBR Green Realtime PCR Master Mix (TOYOBO, Osaka, Japan) reagent following the manufacturer’s instructions. The primer pairs used are shown in Table S2. Each sample analysis was repeated three times.

### 2.9. Subcellular Localization of MpUGTs

The subcellular site of the *Mp*UGTs was inferred from the fluorescence of the GFP fusion plasmid in transiently transformed *N. tabacum* leaves. The *Mp*UGTs ORF sequences lacking their stop codon were amplified using the corresponding primer pair listed in Table S3, and the amplicons were then introduced into pGWB5 (plant green fluorescent overexpression vector with 35S as promoter to promote GFP signal) using the Gateway cloning technique. The validated recombinant plasmids were transferred into *Agrobacterium tumefaciens* GV3101 using the freeze-thaw method [35]. The *A. tumefaciens* were reactivated in YEP medium until OD600 reached 0.8. Then the bacteria were collected and resuspended in a solution containing 10 mM MES-KOH (pH 5.7), 10 mM MgCl_2_ and 15 μM acetosyringone. Similarly, we also cultivated *A. turnefaciens* that contained the gene encoding silencing suppressor protein p19 or an empty vector. The target gene (or empty vector) and p19 gene were mixed 1:1 to infect tobacco epidermal leaf cells. GFP signals were detected using a confocal laser scanning microscope (LSM700, Zeiss, New York, USA). The bandpass filters used were 495–570 nm (GFP) and 650–760 nm (chlorophyll).

### 2.10. Homology Modeling and Molecular Docking of MpUGTs

The three-dimensional structure models of *Mp*UGT737B1 and *Mp*UGT741A1 were constructed using the crystal structure of *Pa*GT2 (PDB code: 6jem) as a template with the SWISS-MODEL server. 

A molecular docking analysis of the *Mp*UGTs with UDP-glucose as the sugar donor and phloretin or lunularin as the acceptor was performed using Maestro software. The localization of UDP-glucose in the *Mp*UGT model was based on the position of UDP-2 fluoro-glucose co-crystallized with *Pa*GT2 [36], followed by docking of different sugar acceptors in the structure. Each complex model with the highest docking score was selected for visualization analysis using PymolWin software.

## 3. Results

### 3.1. Selection and Phylogenetic Analysis of Candidate UGTs from M. polymorpha

Two putative flavonoid UGTs were identified from the genomes of *M. polymorpha* and were named as *Mp*UGT737B1 and *Mp*UGT741A1 by the UGT Nomenclature Committee. The full length cDNAs encoding these *Mp*UGTs were amplified using the cDNAs derived from the thallus of *M. polymorpha*. The open reading frames (ORFs) of *UGT737B1* (PTQ47498) and *UGT741A1* (PTQ40596) were 1443 and 1392 bp, respectively. They encoded polypeptides of 480 and 463 amino acid residues. 

**Figure 1:**
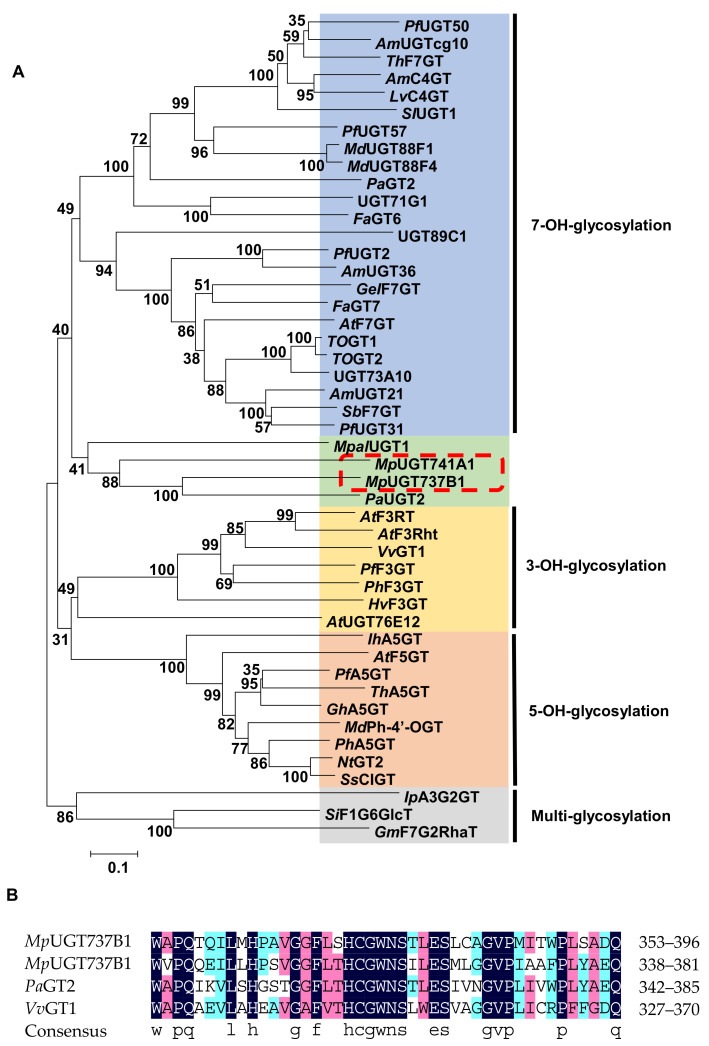
Phylogenetic analysis and sequence alignment of *M. polymorpha* UGTs. (**A**) Phylogenetic tree of *Mp*UGTs. The sequences were aligned using the ClustalW algorithm, based on the neighbor-joining method. (**B**) PSPG boxes of *Mp*UGTs, *Pa*GT2 and *Vv*GT1.

Phylogenetic analysis was performed using these *Mp*UGTs and other flavonoid glycosyltransferases (Figure 1A). The resulting tree was divided into clusters encoding 3-*O*, 5-*O*, 7-*O* glycosyltransferases and diglycoside/disaccharide chain glycosyltransferases. *Mp*UGT737B1 and *Mp*UGT741A1 were in a separate cluster at the root of the 7-*O*-glycosyltransferase cluster. The homology alignment showed about 30% identity of amino acid sequences between *Mp*UGT737B1 and *Mp*UGT741A1, whereas their similarity with *Vitis vinifera* GT was only about 20%. However, the sequence alignment with *Vv*GT1 and *Pa*GT2 showed that the *Mp*UGTs have a conservative PSPG (plant secondary product UGT consensus sequence) motif containing 44 amino acid residues in the C-terminal region (Figure 1B). The last glutamine (Q) residue in the PSPG motif is considered to give the enzyme the specificity of UDP-glucose as the sugar donor. It is worth noting that two *Mp*UGTs possess this Q, suggesting that they probably use UDP-glucose as a sugar donor.

### 3.2. In Vitro Functional Characterization of Recombinant MpUGTs

To test the biochemical function of the *Mp*UGTs, the expressed recombinant proteins were purified and analyzed using SDS-PAGE (Figure S1). Enzyme assays were carried out with a range of flavonoids, bibenzyls and phenylpropanoids as sugar acceptors and UDP-glucose as the sugar donor. The reaction products were analyzed by reversed-phase HPLC and the conversion rates were calculated (Table 1).

**Figure 2:**
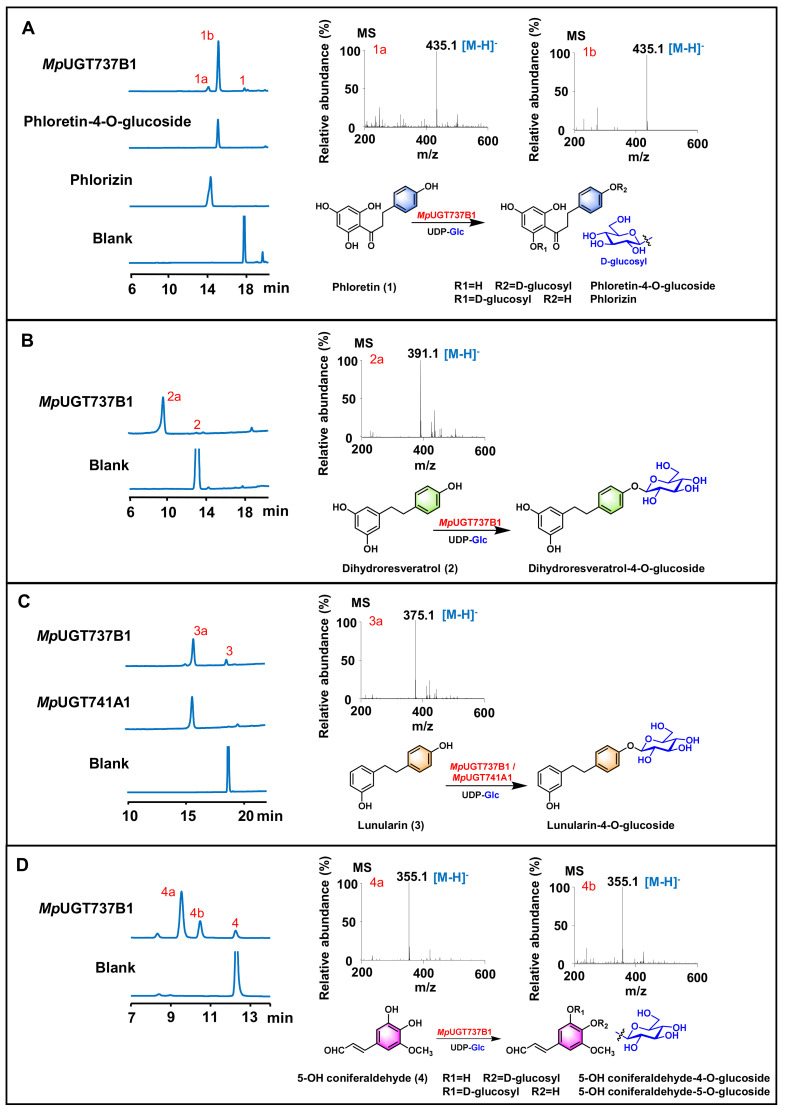
In vitro assays of recombinant *Mp*UGT737B1 and *Mp*UGT741A1 using UDP-glucose as the sugar donor. The HPLC analysis, product LC-MS analysis and catalytic reaction formula of the enzyme catalyzed reaction of *Mp*UGT737B1 with (**A**) phloretin, (**B**) dihydroresveratrol and (**D**) 5-OH coniferaldehyde as the substrate. The HPLC analysis, product LC-MS analysis and catalytic reaction formula of the enzyme catalyzed reaction of *Mp*UGT737B1 and *Mp*UGT741A1 with (**C**) lunularin as the substrate.

*Mp*UGT737B1 showed high activity for dihydrochalcone phloretin and bibenzyls dihydroresveratrol and lunularin (Figure 2A–C). However, *Mp*UGT737B1 could not accept flavones, flavonols and flavanones as substrates (Table 1). *Mp*UGT737B1 converted phloretin to two products, and the minor peak was identified as phlorizin by comparing the retention time with the standards (Figure 2A). However, the retention time of the main product did not match the existing reference standards for trilobatin. In order to identify the unknown peak, a preparative-scale reaction was performed to produce the glycosylated product, and finally about 5.7 mg of product was obtained. The product was identified as phloretin-4-*O*-β-d-glucoside by comparing NMR, HSQC and ^1^H-^1^H COZY spectrum with reported data [37]. In addition, the carbon-hydrogen coupling constant ^1^*J*_C-1,H-1_ = 160~165 Hz of the terminal carbon in the HSQC spectrum also proves that the product is β-D configuration (Figures S2–S4 and Table S4). Accordingly, we speculated that when dihydroresveratrol and lunularin were used as substrates, *Mp*UGT737B1 might also catalyze the glycosylation of the para hydroxyl group in the aromatic ring to produce the corresponding 4-*O*-glucosides (Figure 2B,C). Interestingly, the *Mp*UGT737B1 was also active against several phenylpropanoids, including 5-OH coniferaldehyde, coniferyl alcohol, caffeoyl aldehyde, coniferaldehyde. *Mp*UGT737B1 could catalyze the production of two glycosides using the substrates with two vicinal phenolic hydroxyl groups (Table 1, Figure 2D and Figure S5). The above results indicate that *Mp*UGT737B1 could efficiently catalyze the glycosylation of dihydrochalcone, bibenzyls and phenylpropanoids. *Mp*UGT741A1 could catalyze the glycosylation of lunularin with UDP-glucose as the sugar donor in vitro with a conversion rate close to 100% (Table 1, Figure 2C). In addition, *Mp*UGT741A1 could only accept flavonoids at very low levels (Table 1). These results indicate that *Mp*UGT741A1 is a highly substrate-specific bibenzyl *O*-glycosyltransferase.

To further explore the sugar donor promiscuity of the *Mp*UGTs, *Mp*UGT737B1 and *Mp*UGT741A1 were assayed with UDP-galactose and UDP-glucuronic acid as sugar donor, respectively. The results indicate that *Mp*UGT737B1 and *Mp*UGT741A1 only accept these two sugar donors as substrates at very low levels (Figure S6). Therefore, both enzymes display sugar donor specificity for UDP-glucose, as predicted by the PSPG motif sequence alignment.

### 3.3. Kinetic Analysis of MpUGT737B1 and MpUGT741A1

*Mp*UGT737B1 exhibited maximum capacity at pH 7.0~7.5 (200 mM Tris-HCl) and 30 °C, while *Mp*UGT741A1 was most active at pH 7.0 (200 mM Tris-HCl) and 25~30 °C. The addition of divalent metal ions did not significantly improve the enzyme activity (Figure S7). 

Under the optimized conditions, the kinetic characteristics of *Mp*UGT737B1 and *Mp*UGT741A1 were analyzed by using UDP-glucose as the sugar donor, phloretin or lunularin as the acceptor substrate of *Mp*UGT737B1 and lunularin as the acceptor substrate of *Mp*UGT742A1. The enzyme kinetic parameters associated with *Mp*UGT737B1 and *Mp*UGT741A1 are summarized in Table 2. As suggested by the initial activity screening (Table 1), *Mp*UGT737B1 had significant activity against dihydrochalcone phloretin (*k_cat_*/*K_m_* of 1244.2 M^–1^ s^–1^). *Mp*UGT741A1 had a high catalytic efficiency for lunularin (*k_cat_*/*K_m_* of 1481.6 M^–1^ s^–1^) and was superior to that of *Mp*UGT737A1 (*k_cat_*/*K_m_* of 846.2 M^–1^ s^–1^) (Table 2).

### 3.4. Bioconversion of Dihydroresveratrol and Phloretin into Their 4-O-Glucosides in E. coli

**Figure 3:**
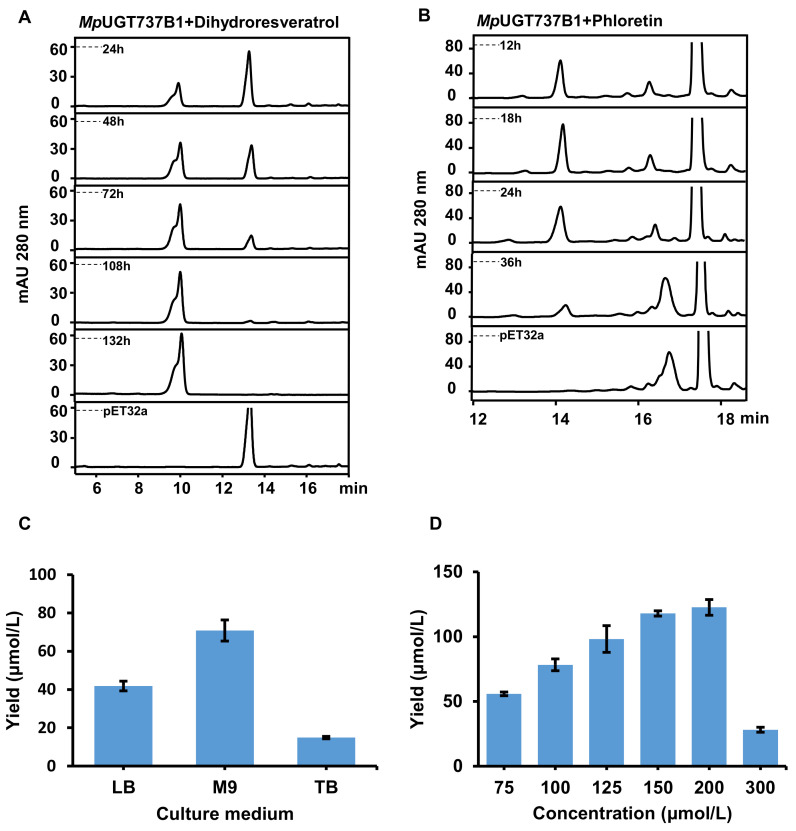
Production of 4-*O*-glucosides by bioconversion using *E. coli* *Mp*UGT737B1. HPLC analysis of recombinant *E. coli Mp*UGT737B1 strain to produce *O*-glucosides when fed with either (**A**) 100 μM dihydroresveratrol or (**B**) 100 μM phloretin. (**C**) The effect of culture medium on the production of phloretin-4-*O*-glucoside. (**D**) The effect of phloretin concentrations on the production of phloretin-4-*O*-glucoside. Three replicates were carried out for each analysis and the error bars indicate the SD.

Wild-type *E. coli* could synthesize endogenous UDP-glucose, which could be utilized as the sugar donor source in the reaction, without addition of purified UDP-glucose. Bioconversion of substrates into their *O*-glucosides was examined using the recombinant *E. coli* MpUGT737B1 strain with dihydroresveratrol and phloretin as the substrates. HPLC analysis results showed that dihydroresveratrol could be completely converted to dihydroresveratrol 4-*O*-glucoside (Figure 3A). When phloretin was added to the medium, it was converted into phloretin-4-*O*-glucoside as the main product, which reached a peak at 18 h, and trace phlorizin was also detected (Figure 3B).

The effects of the media and substrate concentration on production were determined with phloretin as the substrate. The results indicated that M9 medium was the most suit-able medium, and the yield was 42.8% higher than that of LB medium (Figure 3C).

To investigate the optimal substrate concentration, we added different concentrations of phloretin (75–300 µM) into the medium for the biotransformation. The results showed that the yield of phloretin-4-*O*-glucoside increased with the increase of phloretin concentration. When the phloretin concentration was 200 µm, the maximum yield reached 122.6 ± 6.1 µmol/L, but the yield decreased rapidly under 300 µm substrate. It was suspected that the substrate concentration was too high and inhibited bacterial growth (Figure 3D). 

### 3.5. Analysis of Gene Expression Patterns after UV Treatment

**Figure 4:**
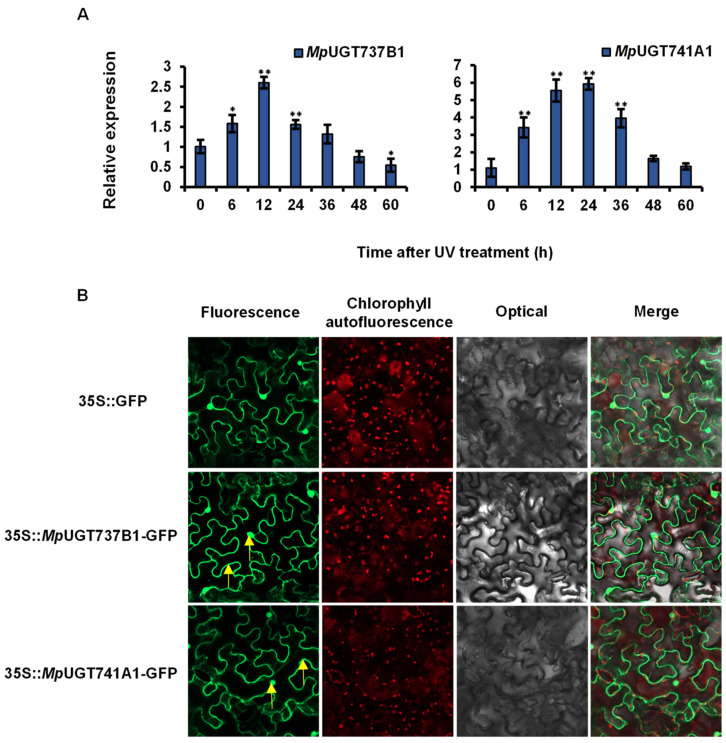
Expression patterns of *Mp*UGTs in response to ultraviolet (UV) light stress (**A**) and sub-cellular localization of *Mp*UGTs (**B**). (**A**) Data are shown in the form mean ± SD (*n* = 3). *, ** Means differ significantly from the level of sample *t* = 0 h at *P* < 0.05 and *P* < 0.01, respectively. (B) The GFP signal appears green and the chlorophyll signal appears red. Yellow arrows show the luminous form of gene localization.

In a previous investigation, the ultraviolet radiation could increase the gene expression of several plant flavonoid UGTs [38]. In the present study, we analyzed the gene expression patterns of the *Mp*UGTs after UV treatment. The results indicate that *Mp*UGT737B1 transcripts in the *M. polymorpha* thallus were induced by UV irradiation with a more than 2.5-fold increase at 12 h and a sharp decrease at 48 h that reached control levels (Figure 4A). The transcripts of *Mp*UGT741A1 also peaked at 12 h, increased by more than 5.5-fold compared with the control, and began to decline after 24 h (Figure 4A). The transcript levels declined to their lowest at 60 h similar to control levels.

### 3.6. Subcellular Localization of MpUGTs

To investigate the *Mp*UGTs subcellular localization, C-terminal green fluorescent protein (GFP) fusion constructs for *Mp*UGT737B1 and *Mp*UGT741A1 were expressed in the leaf epidermal cells of tobacco (*N. tabacum*) using *Agrobacterium*-mediated transformation. A GFP signal was detected in both the cytoplasm and the nucleus expression for either *Mp*UGT737B1 or *Mp*UGT741A1 (Figure 4B). This indicates that *Mp*UGT737B1 and *Mp*UGT741A1 mainly exist as soluble proteins in the nucleus and cytoplasm. 

### 3.7. Homology Modeling and Docking Analysis

To explore the underlying molecular basis of *Mp*UGTs specificity, we selected *Pa*GT2 as the template for homology modeling and molecular docking (Figure S8A). Based on the docking results of *Mp*UGT737B1 and *Mp*UGT741A1, we found that most of the residues that form interactions with the sugar donor were conserved (Q357/W375/N376/E380/Q397 in *Mp*UGT737B1, Q342/W360/N361/E365/Q382 in *Mp*UGT741A1 and Q346/W364/N365/E369/Q386 in *Pa*GT2) (Figure S9).

The sugar receptors were located in the C-terminal cavity of the protein in the *Mp*UGTs. In *Mp*UGT737B1, the MMGBSA binding energy of phloretin were −50.30 kcal/mol, and the 4-OH of phloretin was closer to UDP-glucose, consistent with the feature that *Mp*UGT737B1 preferentially catalyzed the formation of phloretin-4-*O*-glucoside (Figure S8A,B). Furthermore, we compared substrate-binding pocket sizes by modeling both *Mp*UGT737B1 and *Mp*UGT741A1 with lunularin, and the binding energies were −39.03 kcal/mol and −50.12 kcal/mol, respectively. Likewise, the 4-OH position of lunularin was closer to UDP-glucose, further demonstrating the selectivity of *Mp*UGT737B1 and *Mp*UGT741A1 for the 4-position of lunularin. The difference was that in the structure of the *Mp*UGT737B1 double-docking complex, the binding pocket around the sugar receptor was relatively wide (Figures S8C and S9D). In *Mp*UGT741A1, the active cavity was narrow and slender, and mostly only small molecules such as lunularin could easily enter (Figures S8D and S9E). Furthermore, we found that the F190, T147 and D396 amino acid positions in the substrate-binding pocket of *Mp*UGT737B1 correspond to Y202, C134 and E381 in *Mp*UGT741A1, respectively, so they might be key amino acids for the specificity of *Mp*UGTs activity (Figure S9D,E).

The results of docking *Mp*UGTs with glycoside products showed that they all have high binding free energies, as the MMGBSA binding energies of *Mp*UGT737B1 with phloretin-4-*O*-glucoside and lunularin-4-*O*-glucoside were −52.87 and −53.70 kcal/mol, respectively, and the *Mp*UGT741A1 with lunularin4-*O*-glucoside was −46.78 kcal/mol (Figure S10).

According to the docking results, the substrate molecules were in a flexible state in the protein cavity, which is the characteristic of single-bond molecules. The double-bond molecules could not easily enter the active cavity due to their rigid structure, just as *Mp*UGT737B1 and *Mp*UGT741A1 had no catalytic activity for resveratrol (Table 1).

## 4. Discussion

*M. polymorpha* is rich in flavonoids and bibenzyls and their glycosides. Flavonoid glycosyltransferases have been extensively investigated in plants, including five flavonoid UGTs identified in liverworts [29,30]. However, the functions of bibenzyl glycosyltransferases have not been characterized. In this study, we cloned and characterized two *Mp*UGTs, *Mp*UGT737B1 and *Mp*UGT741A1. Phylogenetic tree analysis showed that the *Mp*UGTs aggregated into a single cluster with two previously characterized liverworts flavonoid glucosyltransferases *Mpal*UGT1 and *Pa*UGT2. *Mpal*UGT1 from *Marchantia paleacea* is a highly specific enzyme acting as a flavonol glucosyltransferase. *Pa*UGT2 was identified from *Plagiochasma appendiculatum* which can accept various flavonoids and flavonols as substrates to generate the corresponding flavonoid 7-*O*-glycosides or flavonol 3-*O*-glycosides. Additionally, it has weak catalytic activity for phloretin and catalyzes the formation of phloridzin. This single cluster was located at the root of flavonoid 7-*O*-glycosyltransferase, indicating that bibenzyl glycosyltransferases and flavonoid 7-*O*-glycosyltransferases have common phylogeny (Figure 1A). Both *Mp*UGTs contain a conserved PSPG motif, which is the characteristic sequence of UGTs (Figure 1B). 

*In vitro* enzymatic assays showed that both *Mp*UGTs exhibited high activity towards bibenzyls. *Mp*UGT741A1 exhibited strict substrate selectivity and could completely convert lunularin into the corresponding glycoside product. *Mp*UGT737B1 accepted phloretin, dihydroresveratrol, lunularin and a series of phenylpropanoids. Structural modeling and docking results revealed that *Mp*UGT737B1 exhibits a broader interspace in the substrate-binding pocket, which may provide greater flexibility and variability for the substrate (Figure S8). Therefore, *Mp*UGT737B1 showed wider substrate selectivity and is active for more substituted substrates such as phloretin and dihydroresveratrol (Figure 2).

Interestingly, *Mp*UGT737B1 catalyzed phloretin to form phloretin-4-*O*-glucoside and phloridzin, which exhibited inhibitory activity against tyrosinase and antioxidant activity [27]. Several previously characterized UGTs converted phloretin to form phloridzin and trilobatin. For example, in Malus plants, MdUGT88F4 and MdUGT88F1 could regulate the conversion of phloretin to phloridzin [39], and MdPh-4′-OGT could efficiently glycosylate phloretin into trilobatin in vitro [24]. Therefore, for the first time, *Mp*UGT737B1 was demonstrated to specifically and effectively catalyze the production of phloretin-4-*O*-glucoside. *Mp*UGT737B1 exhibited substrate promiscuity; however, it could not accept the common flavonoids (flavones and flavonols). *Mp*UGT737B1 could convert phloretin to phloretin-4-*O*-glucoside, which is probably due to the similar structure between phloretin and bibenzyls. The modeling results indicated that *Mp*UGT737B1 has the potential to preferentially glycosylate the 4-OH position of the compound. 

*Mp*UGT737B1 could also catalyze the formation of phenylpropanoid glycosides. In a previous investigation, glycosylated phenylpropanoids showed antifungal, anti-inflammatory, and anti-melanin effects in vitro [40], and were also involved in the resistance of plants to abiotic stress [17,18]. Arabidopsis plants produced the glycosylated coumarin scopolin and monolignol coniferin when they were submitted to oxidative stress [40]. There were a few glycosyltransferases of phenylpropanoids that had been characterized and reported. Two glycosyltransferases with catalytic activity for phenylpropanoids had been identified in *Arabidopsis thaliana*. It was reported that UGT72E2 from *A*. *thaliana* could glycosylate aldehydes, coniferyl and sinapyl alcohols, while UGT72E1 was specific for sinapaldehyde and coniferaldehyde [41,42]. *Mp*UGT737B1 characterized in liverworts in the present investigation showed substrate promiscuity and could be used for enzyme catalysis to prepare these glycosylated products. 

As glycosylation plays an important role in plant defense and stress tolerance, we analyzed the transcription level under UV irradiation. UV irradiation was considered to cause oxidative damage [43]. The results indicate that the expression level of all the *Mp*UGTs increased considerably after UV treatment compared with the control. It was also demonstrated that ultraviolet radiation can affect the expression of key genes in the biosynthesis of glycoside products, and it is speculated that *Mp*UGTs may respond to environmental stress and have an antioxidant effect on plant defense. In a previous investigation, it was reported that *O*-glycosides play a positive role in plant UV-B protection. OsUGT707A2 and OsUGT706D1 overexpression plants survived with green leaves, while the wild-type plants became dramatically withered after UV-B irradiation [44]. 

## 5. Conclusions

This study identified and functionally characterized two *Mp*UGTs from the basal land plant *M. polymorpha*. *Mp*UGT737B1 was demonstrated to glycosylate dihydrochalcone phloretin, bibenzyls (dihydroresveratrol and lunularin) and phenylpropanoids to form glucosides with antioxidant activity. In particular, *Mp*UGT737B1 could act at the 4-*O* position of phloretin to produce the phloretin-4-*O*-glucoside. *Mp*UGT741A1 showed substrate specificity to lunuralin, and the conversion rate was close to 100%. This is the first characterization of bibenzyl glycosyltransferases, which will enrich the understanding of the key enzymes in the biosynthesis of various glycosides and the structural diversification of bibenzyls in liverworts, promoting the progress of the in vitro biosynthesis of glycoside products.

## Figures and Tables

**Table 1:** Conversion rates of substrates catalyzed by *Mp*UGTs with UDP-glucose as the sugar donor.

	Substrate	*Mp*UGT737B1	*Mp*UGT741A1
Flavones	apigenin	trace ^a^	ND ^b^
	luteolin	ND	trace
	chrysoeriol	trace	ND
Flavonols	quercetin	ND	ND
	kaempferol	ND	ND
	isorhamnetin	ND	ND
Flavanones	naringenin	trace	10.98 ± 0.56
	hesperetin	ND	trace
	liquiritigenin	ND	trace
	pinocembrin	trace	trace
Chalcones	isoliquiritigenin	ND	ND
Isoflavones	genistein	ND	ND
Dihydrochalcone	phloretin	95.29 ± 1.49 ^c^	ND
Bibenzyls	lunularin	90.38 ± 2.32	99.66 ± 0.48
	lunularic acid	ND	ND
	dihydroresveratrol	98.84 ± 1.65	ND
Stilbenes	resveratrol	ND	ND
Coumarins	esculetin	ND	ND
Phenylpropyl	caffeic acid	ND	ND
	caffeoyl aldehyde	62.40 ± 1.19	trace
	coniferaldehyde	70.37 ± 1.32	ND
	coniferyl alcohol	74.45 ± 2.25	ND
	5-OH coniferyl alcohol	ND	ND
	5-OH coniferaldehyde	90.39 ± 0.36	ND
	sinapaldehyde	14.78 ± 0.21	ND
	sinapyl alcohol	ND	ND

^a^ Minor peak that cannot be integrated. ^b^ No product detected. ^c^ Conversion rates (%) ± STDEV.

**Table 2:** Kinetic parameters of the recombinant *Mp*UGT737B1 and *Mp*UGT741A1.

Enzyme	Substrate	*K_m_* (μM)	*V_max_*	*k_cat_*	*k_cat_/K_m_*
			(nmol mg^−1^ min^−1^)	(s^−1^)	(M^−1^ s^−1^)
	Phloretin	50.2 ± 10.6	70.0 ± 5.5	0.062 ± 0.005	1244.2
*Mp*UGT737B1	Dihydroresveratrol	45.6 ± 9.7	100.3 ± 6.9	0.089 ± 0.006	1960.4
	Lunularin	39.3 ± 7.5	37.3 ± 2.2	0.033 ± 0.002	846.2
*Mp*UGT741A1	Lunularin	89.2 ± 17.6	150.4 ± 12.3	0.124 ± 0.012	1504.6

## Data Availability

Data is contained within the article and Supplementary Materials.

## Supplementary Materials

The following supporting information can be downloaded at: https://www.mdpi.com/article/10.3390/antiox11040735/s1, Table S1: Primers for cloning the full length cDNAs.; Table S2: Primers for qRT-PCR.; Table S3: Primers for GFP fusions.; Table S4: ^1^H NMR spectral data of phloretin-4-*O*-glucoside.; Figure S1: SDS–PAGE separation of recombinant *Mp*UGT737B1 and *Mp*UGT741A1. M: molecular mass standards; Lanes 1, 2, 3: pET32a purified protein (empty vector control), *Mp*UGT737B1 purified protein, *Mp*UGT741A1 purified protein.; Figure S2: The ^1^H NMR spectrum of phloretin-4-*O*-glucoside in methanol-*d*_4_ (400 MHz). ^1^H NMR (Methanol-*d4*, 400 MHz): δ = 2.90 (2H, m, H-α), 3.27 (2H, m, H-β), 3.34–3.90 (6H, m, Glc), 4.87 (1H, overlapped, G-1), 5.80 (2H, s, H-3′/H-5′), 7.01(2H, d, J = 8.4 Hz, H-3/H-5), 7.15 (2H, d, J = 8.4 Hz, H-2/H-6).; Figure S3: The HSQC spectrum of phloretin-4-*O*-glucoside in methanol-*d*_4_ (400 MHz).; Figure S4: The ^1^H-^1^H COSY spectrum of phloretin-4-*O*-glucoside in methanol-*d*_4_ (400 MHz).; Figure S5: HPLC analysis of the products generated by the proteins of *Mp*UGT737B1 using phenylpropanoid. The enzymatic reaction uses coniferyl alcohol (A), caffeoyl aldehyde (B), coniferaldehyde (C) and sinapaldehyde (D) as substrates.; Figure S6: Functional assays of *Mp*UGTs recombinant proteins using UDP-galactose and UDP-glucuronic acid as the donor. (A) Functional assays of recombinant *Mp*UGT737B1 using phloretin as the acceptor. (B) Functional assays of recombinant *Mp*UGT741A1 using lunularin as the acceptor.; Figure S7: Effects of reaction pH, temperature and divalent metal ions on the activity of *Mp*UGT737B1 (A) and *Mp*UGT741A1 (B). Phloretin was used as the acceptor and UDP-glucose was used as the sugar donor for *Mp*UGT737B1. Lunularin was used as the acceptor and UDP-glucose was used as the sugar donor for *Mp*UGT741A1.; Figure S8: The three-dimensional crystal modeling and molecular docking analysis of *Mp*UGT737B1 and *Mp*UGT741A1 protein. (A) The structural model of *Mp*UGT737B1 docked with UDP-glucose and phloretin. (B) The substrate-binding pocket in a model of *Mp*UGT737B1 docking with phloretin (Phl). (C) The substrate-binding pocket in a model of *Mp*UGT737B1 docking with lunularin (LN). (D) The substrate-binding pocket in a model of *Mp*UGT741A1 docking with lunularin.; Figure S9: Substrate binding sites in protein 3D structures. (A) Substrates lunularin and UDP-glucose in the structure of *Mp*UGT737B1 protein and amino acid residues that hydrogen bond with the UDP-sugar donor. (B) Substrates lunularin and UDP-glucose in the structure of *Mp*UGT741A1 protein and amino acid residues that hydrogen bond with the UDP-sugar donor. (C) Substrates in the structure of *Pa*GT2 protein (PDB: 6jem) and amino acid residues that hydrogen bond with the UDP-sugar donor. (D) Amino acids residues in the 4Å range around the lunularin in the *Mp*UGT737B1 protein structure and (E) *Mp*UGT741A1 protein structure. Hydrogen bonds are represented by red dotted lines.; Figure S10: Molecular docking analysis of *Mp*UGTs with glycoside products. (A) Molecular docking analysis of *Mp*UGT737B1 with phloretin-4-*O*-glucoside. (B) Molecular docking analysis of *Mp*UGT737B1 with lunularin-4-*O*-glucoside. (C) Molecular docking analysis of *Mp*UGT741A1 with lunularin-4-*O*-glucoside.
