# Supplementary material for: Identification and Characterization of Two Bibenzyl Glycosyltransferases from the Liverwort Marchantia polymorpha
Source: Antioxidants (Basel). 2022 Apr 8;11(4):735. doi: 10.3390/antiox11040735 (PMC9025568; doi:10.3390/antiox11040735)
Supplement: Supplementary file 1 [file antioxidants-11-00735-s001.zip › antioxidants-1623560-SI.pdf]

antioxidants

MDPI

Identification and Characterization of Two Bibenzyl Glycosyltransferases from the Liverwort *Marchantia polymorpha*

Supplementary Materials

Table S1 Primers for cloning the full length cDNAs.

Primer Name	Accession Number	Primer Sequences (5′ to 3′)
*Mp*UGT737B1-BamHI-F	PTQ47498	CGGGATCCCATGGAGTTGACGAACGGGAC
*Mp*UGT737B1-*Not*I-R	PTQ47498	ATAAGAATGCGGCCGCTTACACCATCACGAGGTCTT
*Mp*UGT741A1-BamHI-F	PTQ40596	CGGGATCCATGGGTTCACATGTGGAGCG
*Mp*UGT741A1-*Not*I-R	PTQ40596	ATAAGAATGCGGCCGCTCATCTAACCCTCTGAACCT

Table S2 Primers for qRT-PCR.

Primer Name	Accession Number	Primer Sequences (5′ to 3′)
*Mp*UGT737B1-RT-F	PTQ47498	AGAGGATAGACATTACAGGC
*Mp*UGT737B1-RT-R	PTQ47498	TTCAGCCACTTCAAACACTC
*Mp*UGT741A1-RT-F	PTQ40596	TGGGCTCGTATTCAACTCCT
*Mp*UGT741A1-RT-R	PTQ40596	CTTTTTTCCACGGGCTTAGT

Table S3 Primers for GFP fusions.

Primer Name	Accession Number	Primer Sequences (5′ to 3′)
*Mp*UGT737B1-GFP-F	PTQ47498	GGGGACAAGTTTGTACAAAAAA-GCAGGCTTAACCATGGAGTTGACGAACGGGAC
*Mp*UGT737B1-GFP-R	PTQ47498	GGGGACCACTTTGTACAAGAAA-GCTGGGTCCACCATCACGAGGTCTTGGA
*Mp*UGT741A1-GFP-F	PTQ40596	GGGGACAAGTTTGTACAAAAAA-GCAGGCTTAACCATGGGTTCACATGTGGAGCG
*Mp*UGT741A1-GFP-R	PTQ40596	GGGGACCACTTTGTACAAGAAA-GCTGGGTCTCTAACCCTCTGAACCTCGG

Table S4 ^{1}H NMR spectral data of phloretin-4-*O*-glucoside.

position	Phloretin-4-*O*-glucoside[a]
	δ_H
2	7.15 d
3	7.01 d
5	7.01 d
6	7.15 d

α-H	2.90 m
β-H	3.27 m
3′	5.80 s
5′	5.80 s
G-1	4.87（overlapped）
G-2-7	3.34–3.90 m

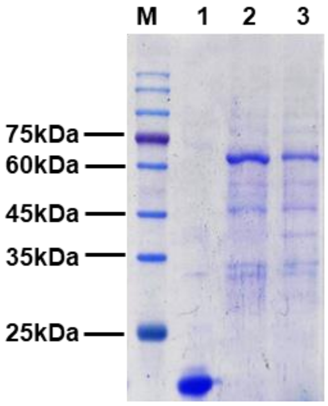

Figure S1. SDS–PAGE separation of recombinant *Mp*UGT737B1 and *Mp*UGT741A1. M: molecular mass standards; Lanes 1, 2, 3: pET32a purified protein (empty vector control), *Mp*UGT737B1 purified protein, *Mp*UGT741A1 purified protein.

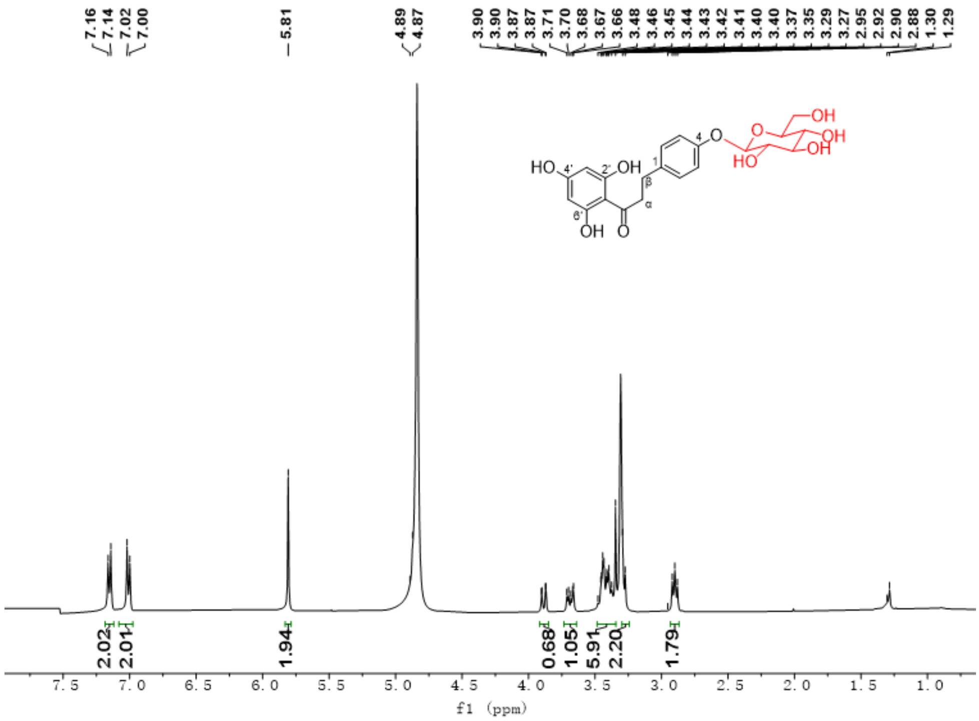

Figure S2. The ^{1}H NMR spectrum of phloretin-4-*O*-glucoside in methanol-d_4 (400 MHz). ^{1}H NMR (Methanol-*d4*, 400 MHz): δ = 2.90 (2H, m, H-α), 3.27 (2H, m, H-β), 3.34–3.90 (6H, m, Glc), 4.87 (1H, overlapped, G-1), 5.80 (2H, s, H-3v/H-5′), 7.01(2H, d, J = 8.4 Hz, H-3/H-5), 7.15 (2H, d, J = 8.4 Hz, H-2/H-6).

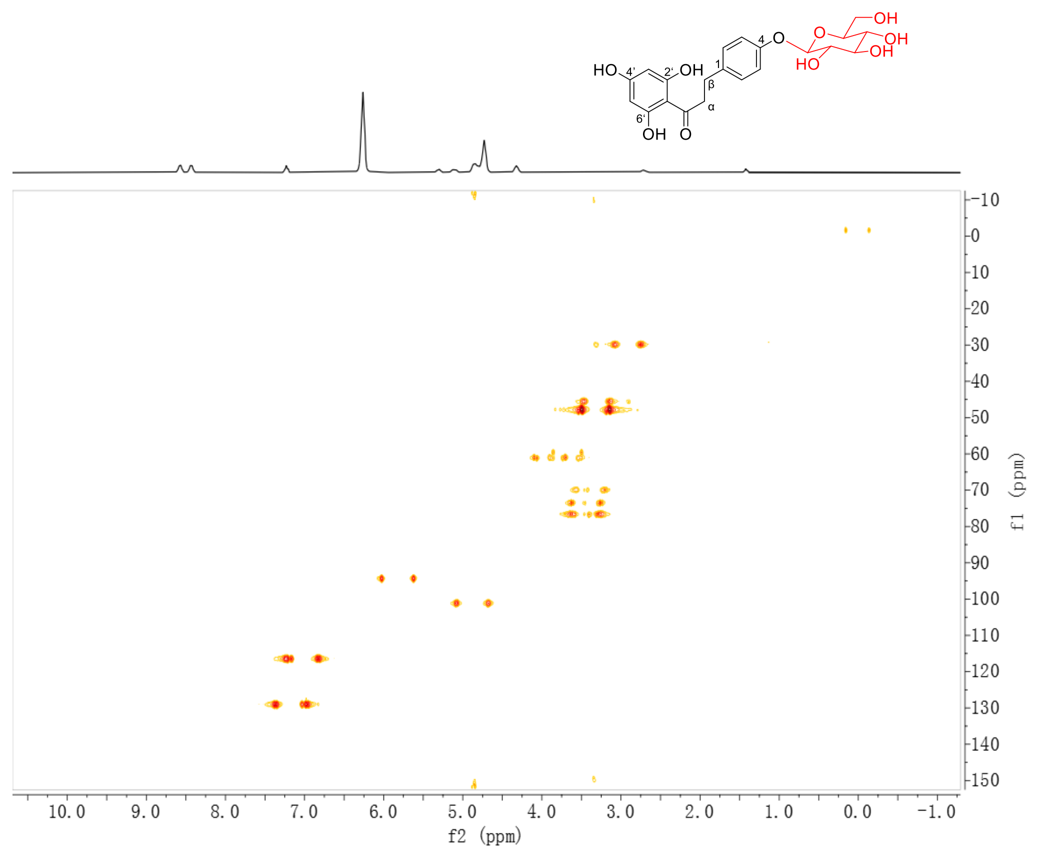

Figure S3. The HSQC spectrum of phloretin-4-*O*-glucoside in methanol-d_4 (400 MHz).

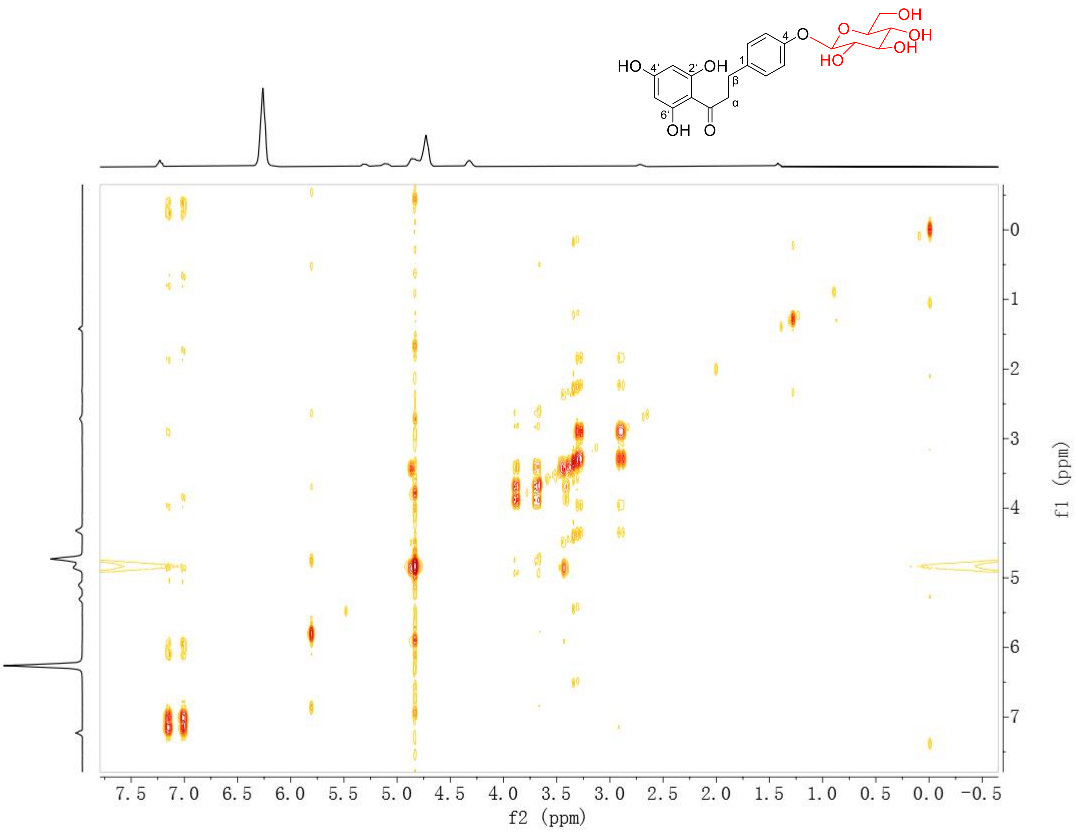

Figure S4. The ^{1}H-^{1}H COSY spectrum of phloretin-4-*O*-glucoside in methanol-d_4 (400 MHz).

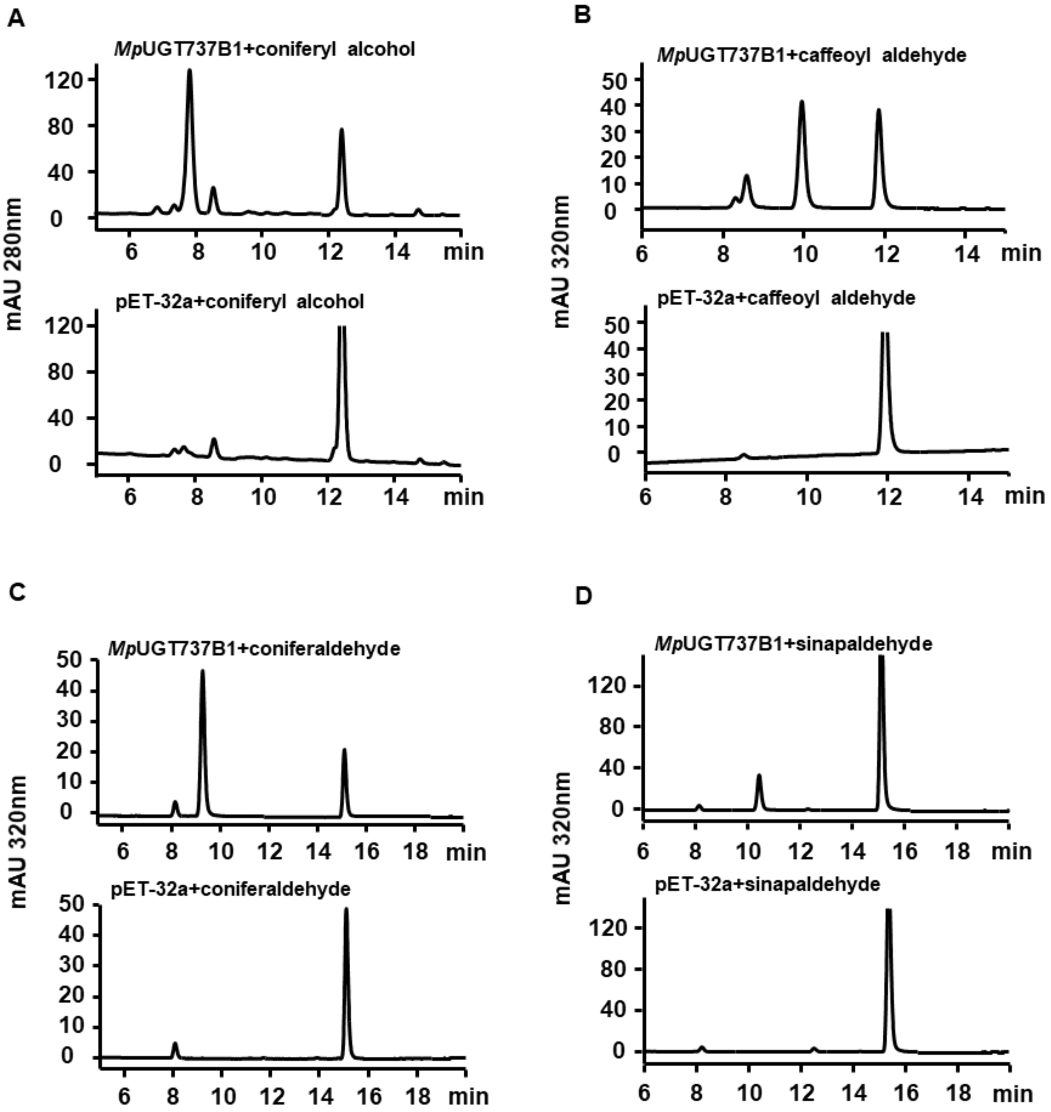

Figure S5. HPLC analysis of the products generated by the proteins of *Mp*UGT737B1 using phenylpropanoid. The enzymatic reaction uses coniferyl alcohol (**A**), caffeoyl aldehyde (**B**), coniferaldehyde (**C**) and sinapaldehyde (**D**) as substrates.

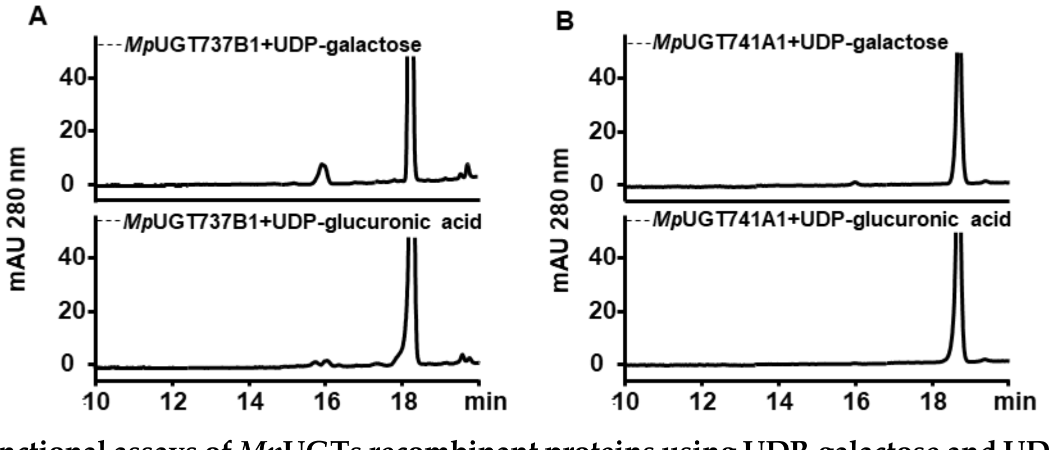

Figure S6. Functional assays of *Mp*UGTs recombinant proteins using UDP-galactose and UDP-glucuronic acid as the donor. (**A**) Functional assays of recombinant *Mp*UGT737B1 using phloretin as the acceptor. (**B**) Functional assays of recombinant *Mp*UGT741A1 using lunularin as the acceptor.

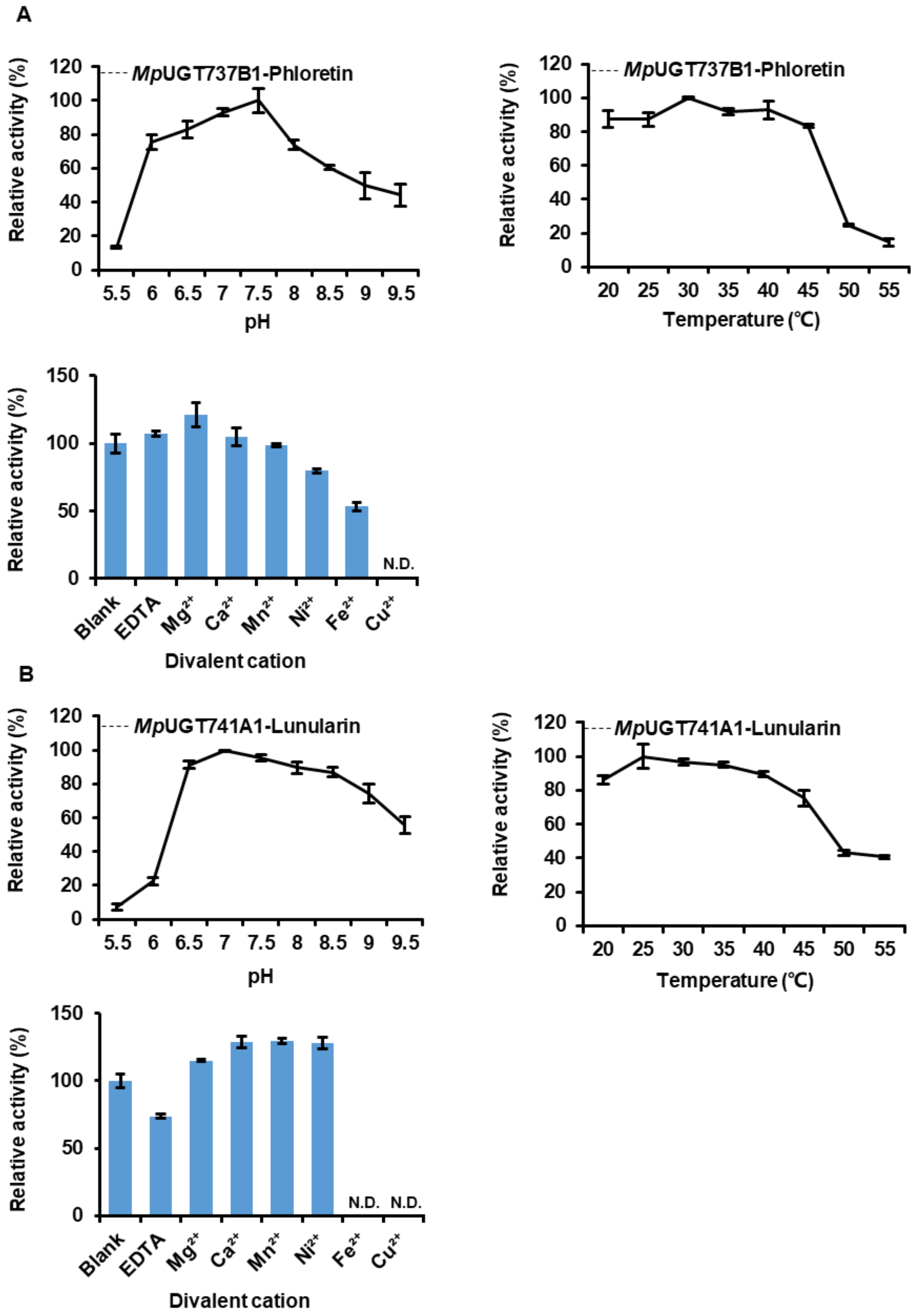

Figure S7. Effects of reaction pH, temperature and divalent metal ions on the activity of *Mp*UGT737B1 (A) and *Mp*UGT741A1 (B). Phloretin was used as the acceptor and UDP-glucose was used as the sugar donor for *Mp*UGT737B1. Lunularin was used as the acceptor and UDP-glucose was used as the sugar donor for *Mp*UGT741A1.

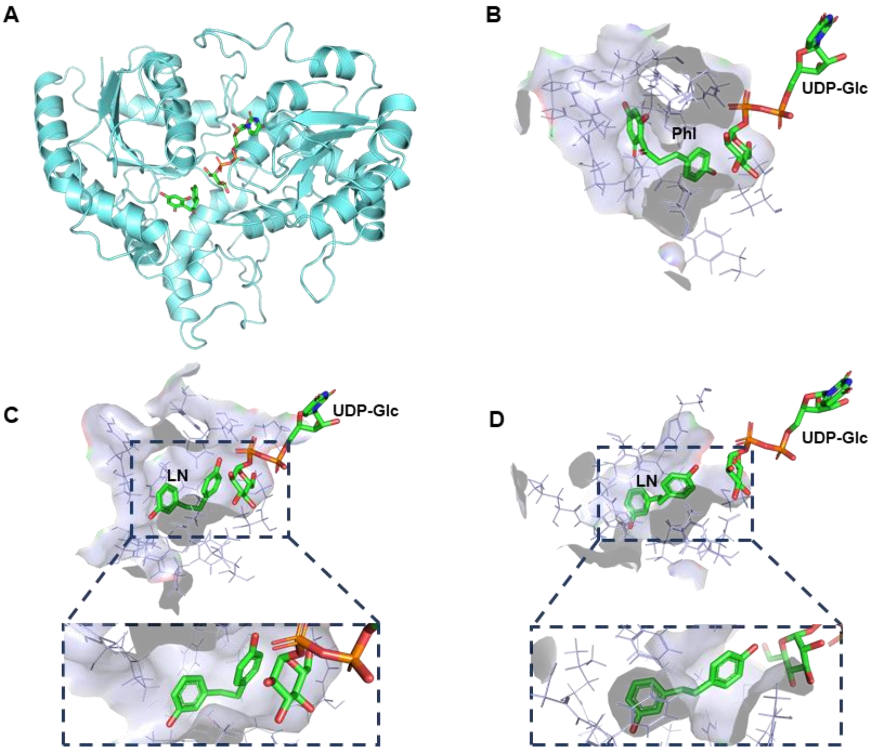

Figure S8. The three-dimensional crystal modeling and molecular docking analysis of *Mp*UGT737B1 and *Mp*UGT741A1 protein. (**A**) The structural model of *Mp*UGT737B1 docked with UDP-glucose and phloretin. (**B**) The substrate-binding pocket in a model of *Mp*UGT737B1 docking with phloretin (Phl). (**C**) The substrate-binding pocket in a model of *Mp*UGT737B1 docking with lunularin (LN). (**D**) The substrate-binding pocket in a model of *Mp*UGT741A1 docking with lunularin.

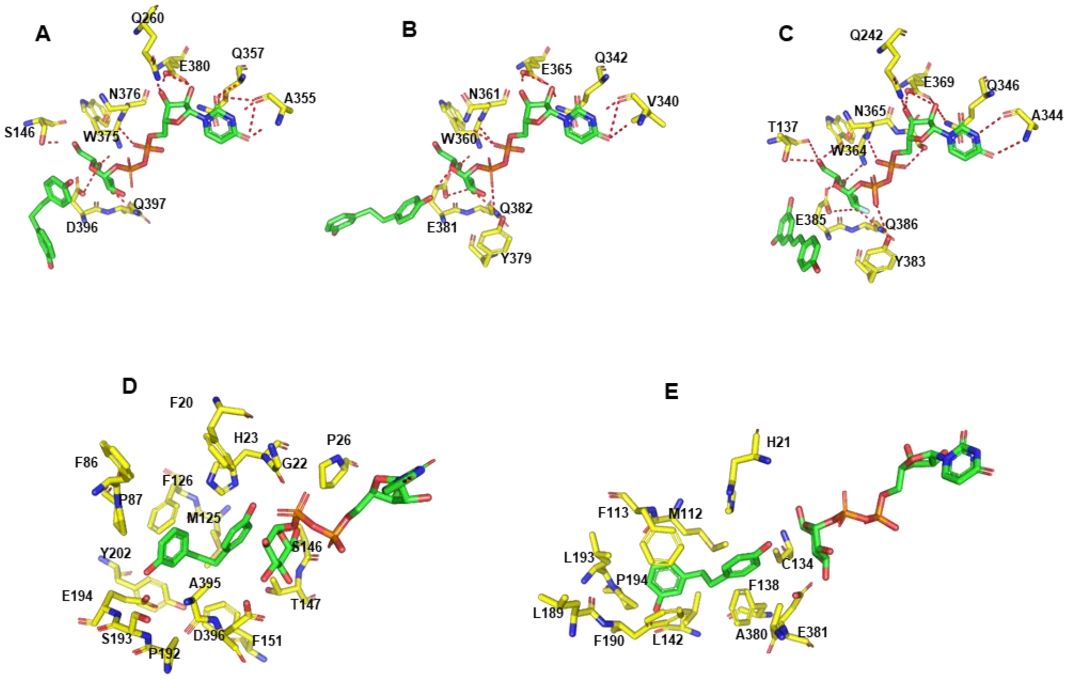

Figure S9. Substrate binding sites in protein 3D structures. (**A**) Substrates lunularin and UDP-glucose in the structure of *Mp*UGT737B1 protein and amino acid residues that hydrogen bond with the UDP-sugar

donor. (**B**) Substrates lunularin and UDP-glucose in the structure of *Mp*UGT741A1 protein and amino acid residues that hydrogen bond with the UDP-sugar donor. (**C**) Substrates in the structure of *Pa*GT2 protein (PDB: 6jem) and amino acid residues that hydrogen bond with the UDP-sugar donor. (**D**) Amino acids residues in the 4Å range around the lunularin in the *Mp*UGT737B1 protein structure and (**E**) *Mp*UGT741A1 protein structure. Hydrogen bonds are represented by red dotted lines.

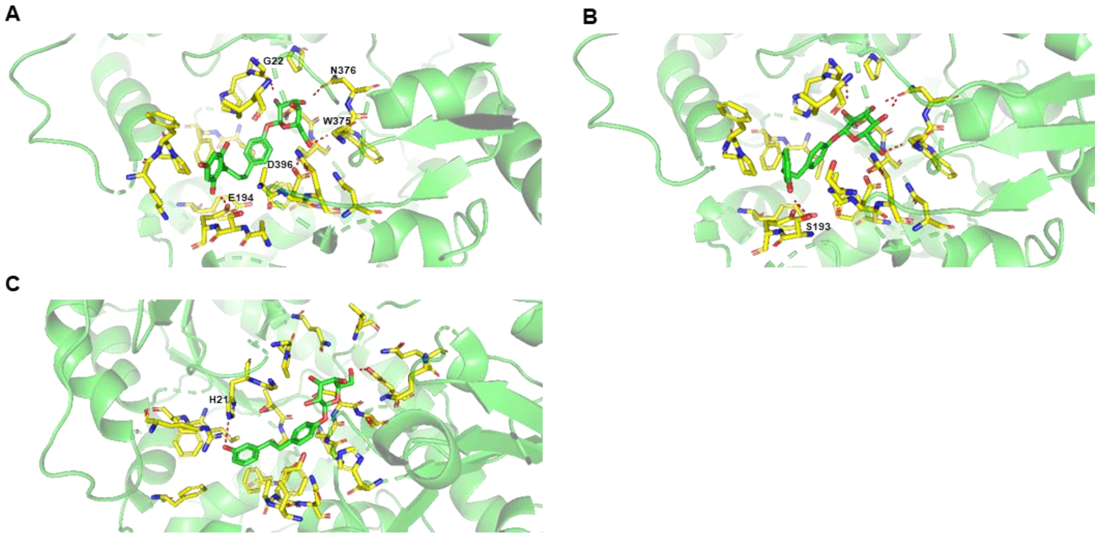

Figure S10. Molecular docking analysis of *Mp*UGTs with glycoside products. (**A**) Molecular docking analysis of *Mp*UGT737B1 with phloretin-4-*O*-glucoside. (**B**) Molecular docking analysis of *Mp*UGT737B1 with lunularin-4-*O*-glucoside. (**C**) Molecular docking analysis of *Mp*UGT741A1 with lunularin-4-*O*-glucoside.
